# Fevogrit, a polyherbal medicine, mitigates endotoxin (lipopolysaccharide)‐induced fever in Wistar rats by regulating pro‐inflammatory cytokine levels

**DOI:** 10.1002/ame2.12472

**Published:** 2024-07-17

**Authors:** Acharya Balkrishna, Sonam Sharma, Vivek Gohel, Rani Singh, Meenu Tomer, Rishabh Dev, Sandeep Sinha, Anurag Varshney

**Affiliations:** ^1^ Drug Discovery and Development Division Patanjali Research Foundation Haridwar India; ^2^ Department of Allied and Applied Sciences University of Patanjali Haridwar India; ^3^ Patanjali UK Trust Glasgow UK; ^4^ Patanjali Yogpeeth Nepal, Mandikhatar Kathmandu Nepal; ^5^ Special Centre for Systems Medicine Jawaharlal Nehru University New Delhi India

**Keywords:** Ayurveda, fever, Fevogrit, lipopolysaccharide, pro‐inflammatory cytokines

## Abstract

**Background:**

Fever is characterized by an upregulation of the thermoregulatory set‐point after the body encounters any pathological challenge. It is accompanied by uncomfortable sickness behaviors and may be harmful in patients with other comorbidities. We have explored the impact of an Ayurvedic medicine, Fevogrit, in an endotoxin (lipopolysaccharide)‐induced fever model in Wistar rats.

**Methods:**

Active phytoconstituents of Fevogrit were identified and quantified using ultra‐high‐performance liquid chromatography (UHPLC) platform. For the in‐vivo study, fever was induced in male Wistar rats by the intraperitoneal administration of lipopolysaccharide (LPS), obtained from *Escherichia coli*. The animals were allocated to normal control, disease control, Paracetamol treated and Fevogrit treated groups. The rectal temperature of animals was recorded at different time points using a digital thermometer. At the 6‐h time point, levels of TNF‐α, IL‐1β and IL‐6 cytokines were analyzed in serum. Additionally, the mRNA expression of these cytokines was determined in hypothalamus, 24 h post‐LPS administration.

**Results:**

UHPLC analysis of Fevogrit revealed the presence of picroside I, picroside II, vanillic acid, cinnamic acid, magnoflorine and cordifolioside A, as bioactive constituents with known anti‐inflammatory properties. Fevogrit treatment efficiently reduces the LPS‐induced rise in the rectal temperature of animals. The levels and gene expression of TNF‐α, IL‐1β and IL‐6 in serum and hypothalamus, respectively, was also significantly reduced by Fevogrit treatment.

**Conclusion:**

The findings of the study demonstrated that Fevogrit can suppress LPS‐induced fever by inhibiting peripheral or central inflammatory signaling pathways and could well be a viable treatment for infection‐induced increase in body temperatures.

## Graphical abstract

Fevogrit, an Ayurvedic medicine indicated for the management of fever, was evaluated for its pharmacological effects in rat model of endotoxin (lipopolysaccharide)‐induced high body temperature. Ultra‐high‐performance liquid chromatography analysis revealed the presence of bioactive phytometabolites in Fevogrit. Fevogrit treatment markedly reduced LPS‐induced fever. Fevogrit also regulated the elevated levels of pyrogenic cytokines both in the serum and in the hypothalamus.

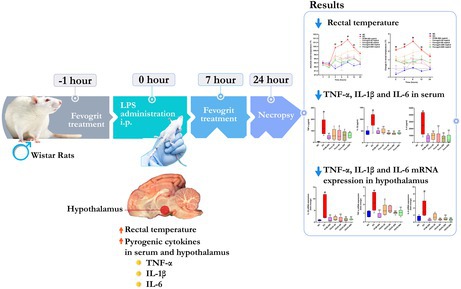

## 1 INTRODUCTION

The development of fever is a physiological reaction of the body to the invasion of infectious or pathogenic agents to neutralize the perceived threat, by a controlled temporary increase of body core temperature above the thermoregulatory set point. The preoptic region of the brain acts as a major thermoregulatory zone of the body mediating core body temperature.^1^ The agents that trigger the induction of the febrile response in the body are termed as pyrogens, mainly characterized as exogenous or endogenous pyrogens. Following the entry of the exogenous pyrogens into the body, the host immune cells secrete pro‐inflammatory cytokines, described as endogenous pyrogens, like tumor necrosis factor‐alpha (TNF‐α), interleukin‐1 Beta (IL‐1β), and interleukin‐6 (IL‐6). These cytokines travel through the blood and reach the organum vasculosum lamina terminalis in the preoptic anterior hypothalamic area of the brain, where they induce the synthesis of prostaglandin E2 (PGE2). PGE2 activates the thermosensitive neurons to increase the temperature set‐point by regulating the processes of body heat production and conservation.^2^ Although fever is a regulated rise in body temperature that benefits the host during illness by inhibiting pathogen replication and enhancing immune‐protective responses, the accompanying sickness behaviors such as anorexia, lethargy, aches and nausea do cause general discomfort to patients. A sustained rise in body temperature can be detrimental to hosts having insufficient metabolic reserves to meet the increased physiological demands during fever and may be potentially more harmful to patients with comorbidities such as impaired lung or cardiac functions.^3^, ^4^ Thus, the treatments that reduce fever relieve the patients from uncomfortable sickness symptoms or potentially associated harmful effects.

The most common stimulus for pathogenic fever is infection by microorganisms. The pyrogenic agent of choice for the induction of experimental fever in animals has been a bacterial endotoxin, lipopolysaccharide (LPS), which is the major component of the outer membrane of gram‐negative bacteria. Systemic injection of the endotoxin into animals initiates a cytokine cascade which in turn interacts with their specific receptors in the hypothalamus to alter the firing rate of thermosensitive neurons and increase the thermal set point.^5^

The most prevalent antipyretic drugs on the market are non‐steroidal anti‐inflammatory drugs (NSAIDs). Among these, acetaminophen (paracetamol) is a widely used antipyretic that inhibits the cyclooxygenase activity and reduces the synthesis of prostaglandins in the brain.^6^ Although effective, NSAIDs have been implicated in increased risk of gastric ulcers, acute kidney injuries and cardiovascular side effects.^7^ Moreover, prolonged use of paracetamol has long been established to be associated with hepatotoxicity at high doses.^8^ Therefore, it is of prime importance to develop a safe and efficient therapeutic antipyretic strategy to combat fever, in general.

The present study was carried out to investigate the efficacy of a novel antifebrile polyherbal medicine, Fevogrit, in a rat model of endotoxin (LPS)‐induced fever. Fevogrit is manufactured by Divya Pharmacy, Haridwar, India by combining a specific portion of six different herbs, described as strong antipyretics and anti‐inflammatories, in the ancient Ayurvedic scriptures. The constituents of Fevogrit include whole plants of *Swertia chirayita* (Chirayata), stems of *Tinospora cordifolia* (Giloy), seeds of *Pongamia pinnata* (Karanj), roots of *Picrorhiza kurroa* (Kutki), whole plant of *Ocimum sanctum* (Tulsi) and thalamus of *Rosa centifolia* (Table 1). We have conducted a detailed phytochemical evaluation of Fevogrit by employing an ultra‐high‐performance liquid chromatography (UHPLC) analytical platform and have identified the essential bioactive constituents present in it. Further, we have conducted an in‐vivo study in order to determine the potential mitigating effect of Fevogrit treatment on the rat model of LPS‐induced fever, at multiple doses. Paracetamol was employed as the reference control compound. Following LPS‐administration, the rectal temperature of animals at different time points over a period of 24 h was determined. The levels of pro‐inflammatory cytokines in serum and their gene expression in hypothalamus were also estimated at the end of the study.

**TABLE 1:** Composition of Fevogrit.

S. no.	Vernacular name	Botanical name	Sanskrit binomial name	Part used	Classical text reference	Page no.	Quantity per tablet (mg)
Extracts of							
1	Chirayata	*Swertia chirayita* (Roxb.) H.Karst.	घूर्णोभदलकम् तिक्तम् Ghūrṇobhadalakam tiktam	Whole plant	Bhavprakash Nighantu, Edition 2010	70	50
2	Giloy	*Tinospora cordifolia* (Willd.) Miers	सप्तशिरिका अरोमपत्रा Saptaśirikā aromapatrā	Stem	Bhavprakash Nighantu, Edition 2010	258	250
3	Karanj	*Pongamia pinnata* (L.) Pierre	करञ्जक: लाजापुष्प: Karañjakaḥ lājāpuṣpaḥ	Seed	Bhavprakash Nighantu, Edition 2010	335	100
4	Kutki	*Picrorhiza kurroa* Royle ex Benth.	कटुका तिक्ता Kaṭukā tiktā	Root	Bhavprakash Nighantu, Edition 2010	67	50
5	Tulsi	*Ocimum tenuiflorum* L	सुमञ्जरिका रामा Sumañjarikā rāmā	Whole plant	The Ayurvedic Formulary of India‐I, Edition 2	496	25
6	Rose	*Rosa centifolia* L	तरुणिका शतपत्रा Taruṇikā śatapatrā	Thalamus	Bhavprakash Nighantu, Edition 2010	475	50

*Note*: Microcrystalline cellulose (MCC) 16 mg, *Acacia Arabica* (Gum Acacia) 10 mg, Croscarmellose sodium 10 mg, and Hydrated magnesium silicate 9 mg, were added to each Fevogrit tablet, in order to generate a stable formulation.

## 2 METHODS

### 2.1 Chemicals and reagents

Fevogrit (Laboratory batch number: PRF/CHI/1222/0359) was sourced from Divya Pharmacy, Haridwar, India. UHPLC grade acetonitrile was procured from Qualigens Mumbai, India. Methanol (Cat. No. M0276), orthophosphoric acid AR grade (Cat. No. O0050) and diethylamine (Cat. No. D0100) were purchased from Rankem, Maharashtra, India. Deionized water was obtained from Milli Q system (Millipore, Billerica, MA, USA). For phytochemical analysis, picroside II (Cat. No. P437640) was procured from Toronto Research Chemicals, Canada, picroside I (Cat. No. 15431) was obtained from Caymann Chemical, Michigan, USA, vanillic acid (Cat. No. 68654) was purchased from Sigma Aldrich, St Louis, MO, USA, cinnamic acid (Cat. No. 29955) was procured from SRL Chemicals, Haryana, India, magnoflorine (Cat. No. CFS202101) and cordifolioside A (Cat. No. CFN95040) were purchased from Chem Faces, Wuhan, Hubei, China. Lipopolysaccharides (LPS) obtained from *Escherichia coli* (0111:B4) for the in‐vivo study were procured from Sigma Aldrich (St Louis, MO, USA). Paracetamol tablets were purchased from Micro labs limited, India and 0.5% methylcellulose was obtained from Loba Chemie Pvt. Ltd, India. ELISA kits for TNF‐α (Cat. No. 558535) and IL‐6 (Cat. No. 550319) were procured from BD Biosciences, USA. IL‐1β (Cat. No. 88‐6010‐22) ELISA kit was obtained from Invitrogen, USA. TRIzol reagent (Cat. No. 15596018) and Verso cDNA synthesis kit (Cat. No. AB‐1453/B) were procured from Thermo Fisher Scientific, USA and PowerUp SYBR Green Master Mix (Cat. No. A25742) was purchased from Applied Biosystems, USA for mRNA expression analysis.

### 2.2 Phytochemical analysis

The identification of the bioactive metabolites of Fevogrit was performed using the Prominence‐XR UHPLC system (Shimadzu, Japan), following a previously reported method for a similar herbal medicine.^9^ The system was equipped with a Quaternary pump (NexeraXR LC‐20AD XR), DAD detector (SPD‐M20 A), Auto‐sampler (Nexera XR SIL‐20 AC XR), Degassing unit (DGU‐20A 5R) and Column oven (CTO‐10 AS VP) (Shimadzu, Japan). The elution was carried out at a flow rate of 1.0 mL/min using gradient elution of mobile phase A (0.1% orthophosphoric acid in water; adjusted to pH 2.5 by diethylamine) and mobile Phase B (acetonitrile). Gradient programming of the solvent system for mobile phase B was set as 5% from 0 to 10 min, 5%–15% from 10 to 25 min, 15%–20% from 25 to 35 min, 20%–35% from 35 to 50 min, 35%–40% from 50 to 60 min, 40%–5% from 60 to 61 min and 5% from 61 to 65 min. The standard stock solutions (1000 μg/mL) of picroside II (potency, 98.0%), picroside I (potency, 100%), vanillic acid (potency, 98.2%), cinnamic acid (potency, 99.7%) magnoflorine (potency, 98.0%) and cordifolioside A (potency, 98.0%) were prepared separately by dissolving accurately weighed standards in methanol. To prepare a 50 μg/mL concentration of working standard solution, 0.05 mL aliquots of each standard solution were mixed and diluted to 1 mL. For preparation of the test solution, 0.5 gm of a powdered sample of Fevogrit was mixed in 10 mL of a water: methanol solution (20:80) and sonicated for 30 min. The solution was then centrifuged at 9000 rpm followed by filtration using 0.45 μm nylon filter and the filtrate was used for the analysis. Subsequently, 10 μL of standard and test solutions were injected separately into Shodex C18‐4E (5 μm, 4.6 × 250 mm) column maintained at 35°C; and the detection of phytochemicals was performed at 270 nm. The results were expressed as μg/mg of Fevogrit.

### 2.3 In‐vivo antipyretic activity of Fevogrit

#### 2.3.1 Animal procurement

Specific Pathogen Free (SPF) male Wistar rats (5–6 weeks) were procured from Hylasco Bio‐Technology Pvt. Ltd, Hyderabad, Telangana, India, a Charles River Laboratories licensed domestic laboratory animal supplier. On receipt, animals were quarantined for 1 week in the Laboratory Animal Facility. The experiments were conducted in accordance with the study protocol (wide number PRIAS/LAF/IAEC‐166) approved by Institutional Animal Ethics Committee of Patanjali Research Institute, Haridwar, India, following the guidelines of the Committee for the Control and Supervision of Experiments on Animals (CCSEA), Department of Animal Husbandry and Dairying, Ministry of Fisheries, Animal Husbandry and Dairying, Govt. of India. Post quarantine, study animals were transferred to an experimental room where they were housed in polypropylene cages at 23 ± 2°C and 60%–70% relative humidity with 12 h light/dark cycle. The animals were provided with a standard pellet diet (Purina lab diet, St. Louis, MO, USA) and sterile filtered water ad libitum throughout the study period.

#### 2.3.2 Dose calculation for in‐vivo experiment

**FIGURE 1:**
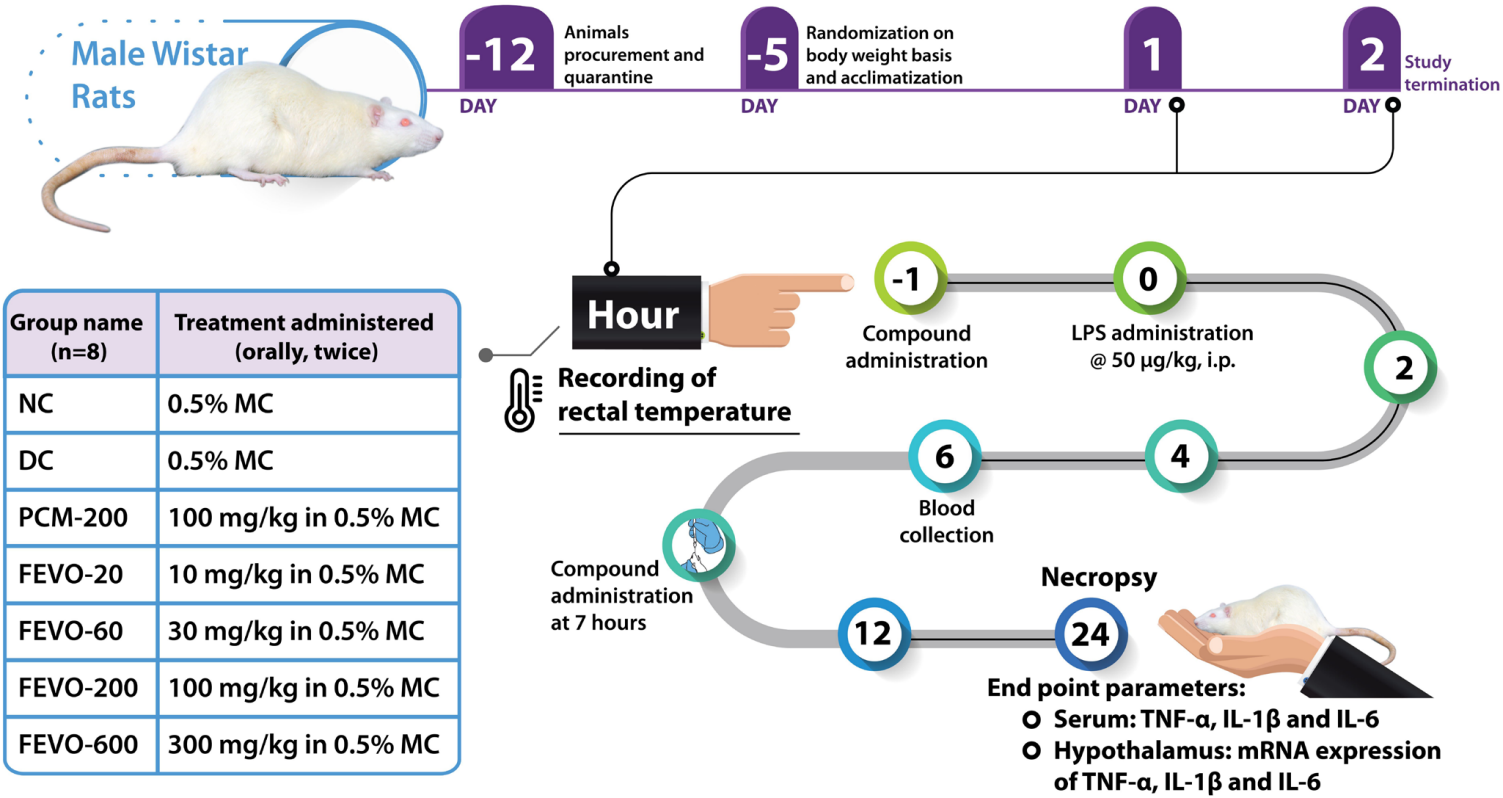
Schematic representation of establishment of endotoxin (LPS)‐induced fever model in Wistar rats. Following quarantine and acclimatization, experimental rats were randomly assigned to seven different groups namely, normal control (NC), disease control (DC), paracetamol (PCM‐200), FEVO‐20, FEVO‐60, FEVO‐200 and FEVO‐600. Animals were orally administered methylcellulose (NC and DC), paracetamol (100 mg/kg, b.i.d.) and four different doses of Fevogrit (10, 30, 100, and 300 mg/kg, b.i.d). One hour after administration of the compounds, the rectal temperature of animals was recorded and an intraperitoneal injection of LPS (50 μg/kg) was administered to animals in all groups except NC, which was injected with an equal volume of normal saline. Rectal temperature was recorded at 2, 4, 6, 12 and 24 h post‐LPS stimulation. At the 6‐h time point, the serum of the animals was collected for measurement of pro‐inflammatory cytokines. At 24 h post‐LPS administration, the hypothalamus of all animals was harvested for estimation of mRNA expression.

The recommended therapeutic dose of Fevogrit for a human of average body weight of 60 kg is 2000 mg/day. Accordingly, the human dose of Fevogrit is 33.33 mg/kg/day. The therapeutic human equivalent dose (mg/kg) for rats was calculated by extrapolating the human dose to animals by multiplying it by a factor of 6.2.^10^ The resultant therapeutic dose for rats was found to be 206 mg/kg/day which was rounded to 200 mg/kg/day and taken as therapeutic dose for the in‐vivo study. Animals were administered Fevogrit twice in 24 h, in four graded doses of 10, 30, 100 and 300 mg/kg as displayed in Figure 1.

#### 2.3.3 Experimental groups

Subsequent to quarantine, the animals were randomized on the basis of their body weight and allocated to the following seven experimental groups consisting of eight animals per group: Normal control – 0.5% methylcellulose (NC), b.i.d., Disease control – 0.5% methylcellulose, b.i.d. (DC), Paracetamol – 100 mg/kg, b.i.d. (PCM), Fevogrit – 10 mg/kg, b.i.d. (FEVO‐20), Fevogrit – 30 mg/kg, b.i.d. (FEVO‐60), Fevogrit – 100 mg/kg, b.i.d. (FEVO‐200) and Fevogrit – 300 mg/kg, b.i.d. (FEVO‐600). The animals received the treatments by oral gavage twice a day (Figure 1).

#### 2.3.4 Induction of fever in animals

LPS solution (10 μg/mL) was prepared in sterile normal saline and warmed to a temperature of 37°C using a water bath. For the induction of fever, 1 h after the oral administration of methylcellulose/Paracetamol/Fevogrit, LPS solution was injected intraperitoneally to the animals of all groups except NC, at a dose of 50 μg/kg in a dose volume of 5 mL/kg.^11^ The animals allocated to NC group were administered an intraperitoneal injection of an equivalent volume of sterile normal warm saline.

#### 2.3.5 Measurement of rectal temperature

Before the initiation of the experiment, rats were accustomed to the polypropylene animal restrainer and to the procedure of temperature recording, for 5 min for 3 consecutive days. On the day of the experiment, the basal rectal temperature of animals was recorded before the oral administration of treatment drugs (−1 h) using a digital thermometer (Dr Odin, China).^12^ Thereafter, rectal temperature was recorded 1 h after the oral treatment, that is, just prior to LPS‐administration (0 h) and at 2, 4, 6, 12 and 24 h post‐LPS administration. The change in rectal temperature at 2, 4, 6, 12 and 24 h was calculated by subtracting the initial temperature recorded at 0 h from the final temperature recorded at the different time points. Additionally, the total pyrexia response over 24 h was determined and presented as the area under the curve (AUC).

#### 2.3.6 Determination of serum levels of pro‐inflammatory cytokines

Six hours post‐LPS administration, blood samples were collected from the retro‐orbital sinus and serum was separated at 3000 × g for 15 min at 4°C using a centrifuge (Thermo Scientific, Germany). The levels of endogenous pyrogens, namely, TNF‐α, IL‐1β and IL‐6, in the separated sera were determined using ELISA kits.^13^, ^14^ The optical density was measured at 450 nm by an EnVision multimode plate reader (PerkinElmer, USA).

#### 2.3.7 Determination of the mRNA expression of pro‐inflammatory cytokines in the hypothalamus

At the end of the study, animals were humanely sacrificed by administering an overdose of thiopentone anesthesia (150 mg/kg) intraperitoneally, 24 h post‐LPS administration. The brain was carefully removed and chilled on plates placed on ice packs. A transverse cut was made to separate the rhombencephalon from the brain. Another transverse cut was made at the level of optic chiasma that delimits the anterior hypothalamus and passes through the anterior commissure. Using the anterior commissure, as the horizontal reference and the line connecting the posterior hypothalamic area and mammillary bodies as the internal limit, the hypothalamus was excised with the utmost care. The hypothalamus was placed in RNA stabilization reagent (RNAprotect, Qiagen, Germany), snap frozen in liquid nitrogen and stored at −80°C for gene expression analysis, by quantitative reverse transcription polymerase chain reaction (qRT‐PCR). Total RNA was extracted from the hypothalamus tissue using TRIzol reagent as per the manufacturer's protocol and was subsequently used to synthesize cDNA using a Verso cDNA synthesis kit. qRT‐PCR analysis was performed in a Real Time PCR System Machine (qTOWER^3^G, Analytik Jena, Germany) using PowerUp SYBR Green Master Mix. qRT‐PCR cycle conditions were as follows: 95°C for 5 min, followed by 40 cycles of denaturation at 95°C for 30 s, annealing at 56–60°C for 30 s, extension at 72°C for 30 s and final extension at 72°C for 5 min. GAPDH was used as a reference gene for normalizing the data. The Ct value of each gene was calculated using the 2^−ΔΔCt^ method, as described previously.^15^ The primer details used for qRT‐PCR are given in Table 2.

**TABLE 2:** Primer details used for qRT‐PCR in this study.

S. no.	Genes	Primer sequence (5′–3′)	Annealing temperature (°C)	Product length (bp)	GenBank accession no.
1	TNF‐α	F‐TACTGAACTTCGGGGTGATCG	56	292	NM_012675.3
		R‐CCTTGTCCCTTGAAGAGAACC			
2	IL‐1β	F‐CACCTCTCAAGCAGAGCACAG	56	79	NM_031512.2
		R‐GGGTTCCATGGTGAAGTCAAC			
3	IL‐6	F‐TCCTACCCCAACTTCCAATGCTC	60	79	NM_012589.2
		R‐GTGGATGGTCTTGGTCCTTAGCC			
4	GAPDH	F‐TGTGAACGGATTTGGCCGTA	60	149	XM_039107008.2
		R‐TGAACTTGCCGTGGGTAGAG			

*Note*: GAPDH was used as the house‐keeping gene.

Abbreviations: F, forward primer; R, reverse primer.

#### 2.3.8 Statistical analysis

All the data are presented as mean ± standard error of mean (SEM). The statistical analysis of absolute and change in rectal temperature data was performed by employing two‐way analysis of variance (ANOVA) followed by Tukey's multiple comparisons test. For absolute rectal temperature at 4 and 6 h, AUC, cytokine analysis and gene expression analysis, one‐way ANOVA was applied followed by Dunnett's multiple comparisons test. All statistical analysis was done using Graph Pad Prism Version 8.0.2 software suite (San Diego, CA, USA). A *p* value <0.05 was considered to be statistically significant.

## 3 RESULTS

### 3.1 Phytometabolite analysis of Fevogrit

**FIGURE 2:**
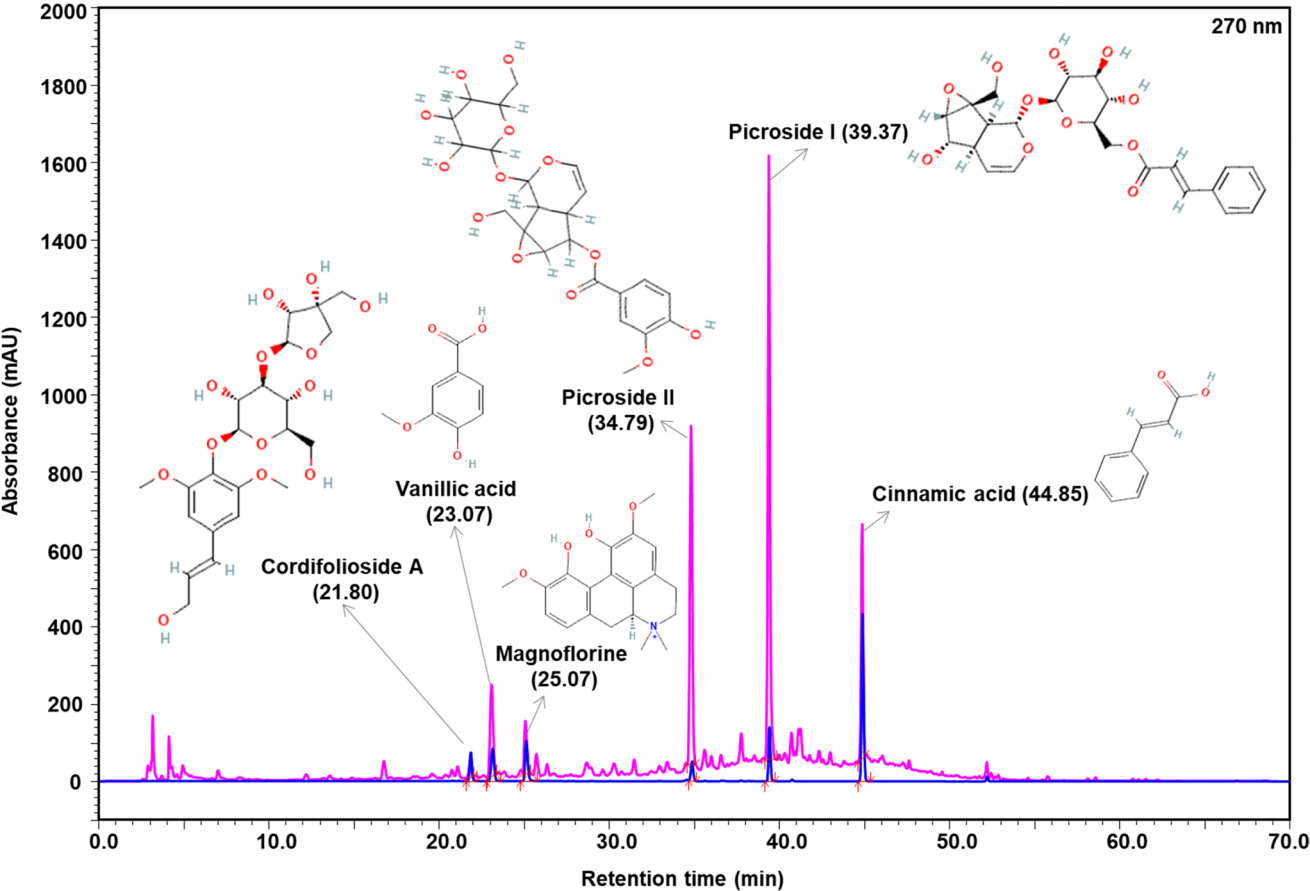
Phytometabolite analysis of Fevogrit by UHPLC. Fevogrit powder (pink line in chromatogram) was analyzed by ultra‐high‐performance liquid chromatography (UHPLC), along with a reference standard mix (chromatogram in blue line). The chromatographic profile of Fevogrit was developed at a wavelength of 270 nm. UHPLC analysis detected the presence of six marker bioactive compounds, namely, picroside II, picroside I, vanillic acid, cinnamic acid, magnoflorine and cordifolioside A, at their respective retention times. The chemical structures of these compounds (sourced from www.PubChem.com) have been depicted alongside the respective identified peaks. The quantification of these phytometabolites against the standard mix is listed in Table 3, along with the observed retention times.

The chromatographic profile of Fevogrit revealed the presence of picroside I, picroside II, vanillic acid, cinnamic acid, magnoflorine and cordifolioside A, as the major bioactive metabolites. The typical UHPLC chromatogram of standards and Fevogrit is shown in Figure 2. The quantitative measurements of these metabolites in Fevogrit are listed in Table 3.

**TABLE 3:** Active phytometabolites present in Fevogrit as per the UHPLC chromatographs shown in Figure 2.

S. no.	Phytometabolite	Retention time (min)	Quantity in Fevogrit (μg/mg)
1	Picroside II	34.79	15.210
2	Picroside I	39.37	12.055
3	Vanillic acid	23.07	9.434
4	Cinnamic acid	44.85	1.522
5	Magnoflorine	25.07	1.295
6	Cordifolioside A	21.80	0.431

### 3.2 Fevogrit demonstrated an antipyretic effect in LPS‐induced rats

**FIGURE 3:**
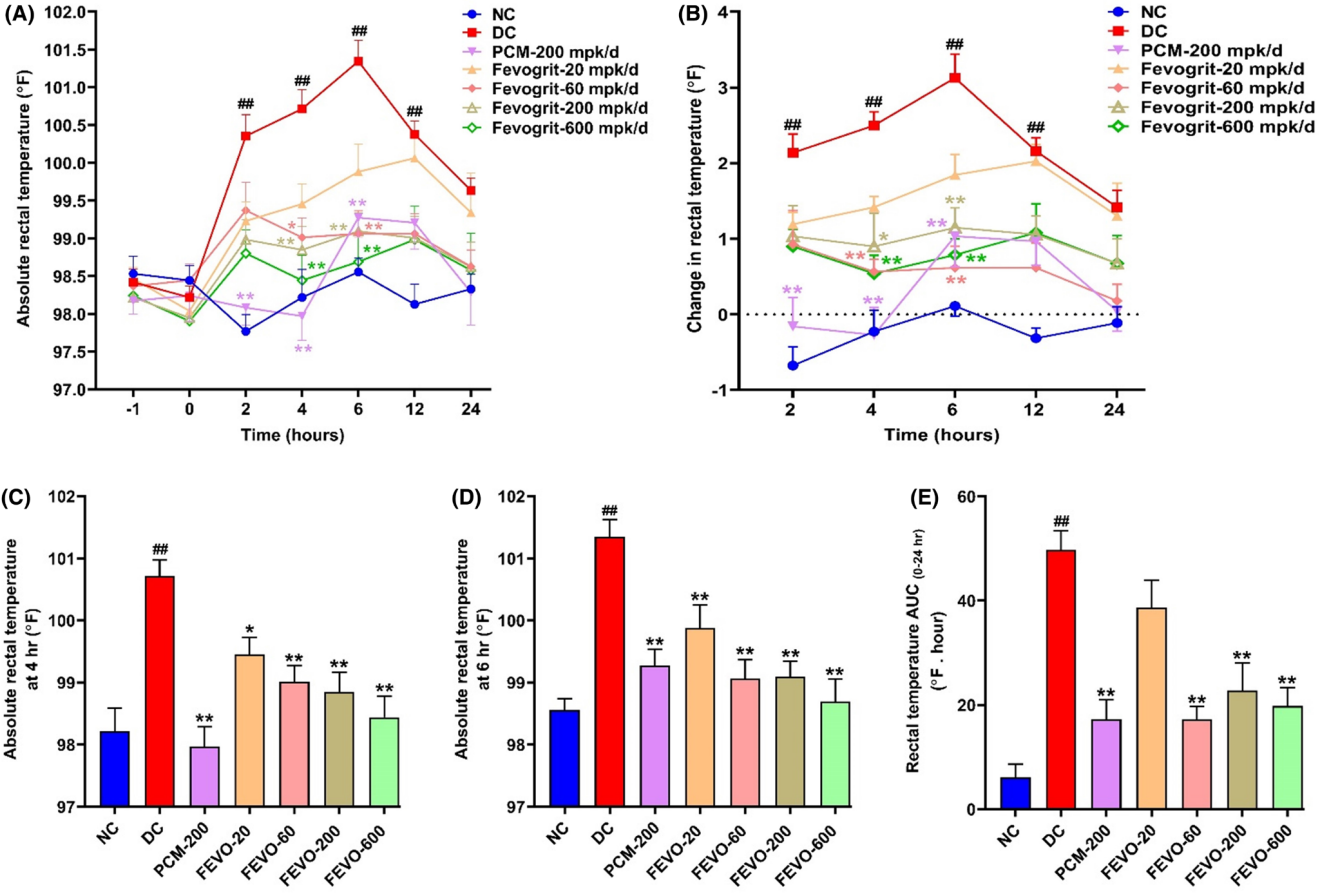
Fevogrit treatment attenuated fever in LPS‐induced Wistar rats. Antipyretic effect of Fevogrit on LPS‐induced elevation in absolute rectal temperature (A) and change in rectal temperature (B), at different time points over a period of 24 h. The bar graphs (C–E) depict absolute rectal temperature at 4 h post‐LPS administration (C), absolute rectal temperature at 6 h post‐LPS administration (D) and total pyrexia response over the experimental period of 24 h (E) as area under the curve (AUC). For the graphs A and B, data were analyzed by two‐way ANOVA followed by Tukey's multiple comparison test whereas for the bar graphs C, D and E, data were statistically analyzed by one‐way ANOVA followed by Dunnett's multiple comparisons test. All the data are expressed as mean ± SEM (*n* = 7 or 8 animals per group). ^##^ *p* < 0.01 vs. NC; *p < 0.05 and **p < 0.01 vs. DC.

As shown in Figure 3A, the LPS challenge in animals (DC group) resulted in a significant (*p* < 0.01) elevation in the rectal temperature at 2, 4, 6 and 12 h post‐LPS administration compared to the NC group. Animals in the DC group exhibited a peak febrile response at 6 h post‐LPS administration when the rectal temperature showed a significant rise of 3.10 °F above the basal temperature, after which it started to decline (Figure 3B). The PCM group demonstrated a peak rise of only 1.04 °F (*p* < 0.01, Figure 3B) at 6 h and a significant reduction at the 2, 4 and 6‐h time points (*p* < 0.01, Figure 3B–D, respectively), relative to the DC group. Interestingly, the FEVO‐20, 60, 200 and 600 groups exhibited a significant suppression of the absolute rectal temperature at 4 and 6 h post‐LPS administration (Figure 3C,D) with increases of 1.80 °F, 0.62 °F (*p* < 0.01), 1.15 °F (*p* < 0.01) and 0.79 °F (*p* < 0.01), respectively, at the 6‐h time point in comparison to the DC group (Figure 3B). Moreover, the rise in rectal temperature in the FEVO‐60, 200 and 600 groups was less than half that of the DC group, at all recorded time points from 2 to 24 h (Figure 3B). Additionally, the total pyrexia response calculated as AUC over the experimental period of 24 h, was found to be significantly decreased in the PCM, FEVO‐60, 200 and 600 groups compared to the DC group (*p* < 0.01, Figure 3E). The pyrexia response was reduced to more than half in the PCM, FEVO‐60, FEVO‐200 and FEVO‐600 groups. However, Fevogrit at a dose of 20 mg/kg/day was less effective in mitigating fever compared to the other treatment groups. Taken together, these findings indicate that Fevogrit treatment has a pronounced suppressive effect on the fever induced by LPS administration in Wistar rats.

### 3.3 Fevogrit attenuated the LPS‐induced increase in pro‐inflammatory cytokines levels

**FIGURE 4:**
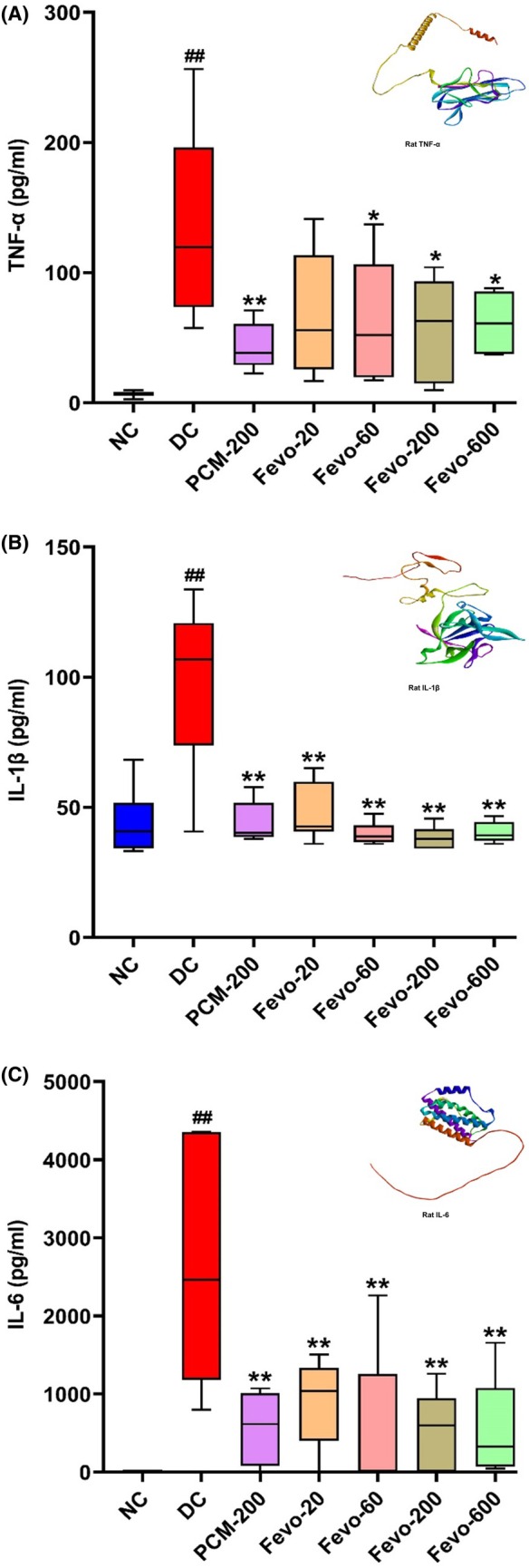
Fevogrit modulated circulatory levels of pro‐inflammatory cytokines in LPS‐induced Wistar rats. The bar graphs (A–C) depict the inhibitory effect of Fevogrit treatment on the serum levels of different pro‐inflammatory cytokines, namely, TNF‐α (A), IL‐1β (B) and IL‐6 (C). Data are expressed as mean ± SEM (*n* = 5–8 animals per group) and were statistically analyzed by one‐way ANOVA followed by Dunnett's multiple comparisons test. ^##^ *p* < 0.01 vs. NC; *p < 0.05 and **p < 0.01 vs. DC.

As pro‐inflammatory cytokines act as the most important endogenous pyrogens, we investigated the serum levels of TNF‐α, IL‐1β and IL‐6 in animals at 6 h post‐LPS administration. As shown in Figure 4, Fevogrit and paracetamol treatment ameliorated the effect of LPS on elevated peripheral levels of TNF‐α, IL‐1β and IL‐6 and exhibited a marked reduction of these endogenous pyrogens (*p* < 0.01, Figure 4A–C). Compared to the DC group, paracetamol significantly reduced the levels of all three evaluated cytokines (*p* < 0.01, Figure 4A–C). The test substance, Fevogrit significantly attenuated the LPS‐induced release of TNF‐α, at doses of 60, 200 and 600 mg/kg/day (*p* < 0.05, Figure 4A) and attenuated the serum levels of IL‐1β and IL‐6 levels to a greater extent at all tested doses (*p* < 0.01, Figure 4B,C) compared to the DC group. These observed changes in cytokine levels reaffirm that LPS‐induced release of endogenous pyrogens is efficiently attenuated with Fevogrit treatment.

### 3.4 Fevogrit mitigated the increased mRNA expression of pro‐inflammatory cytokines in hypothalamus

**FIGURE 5:**
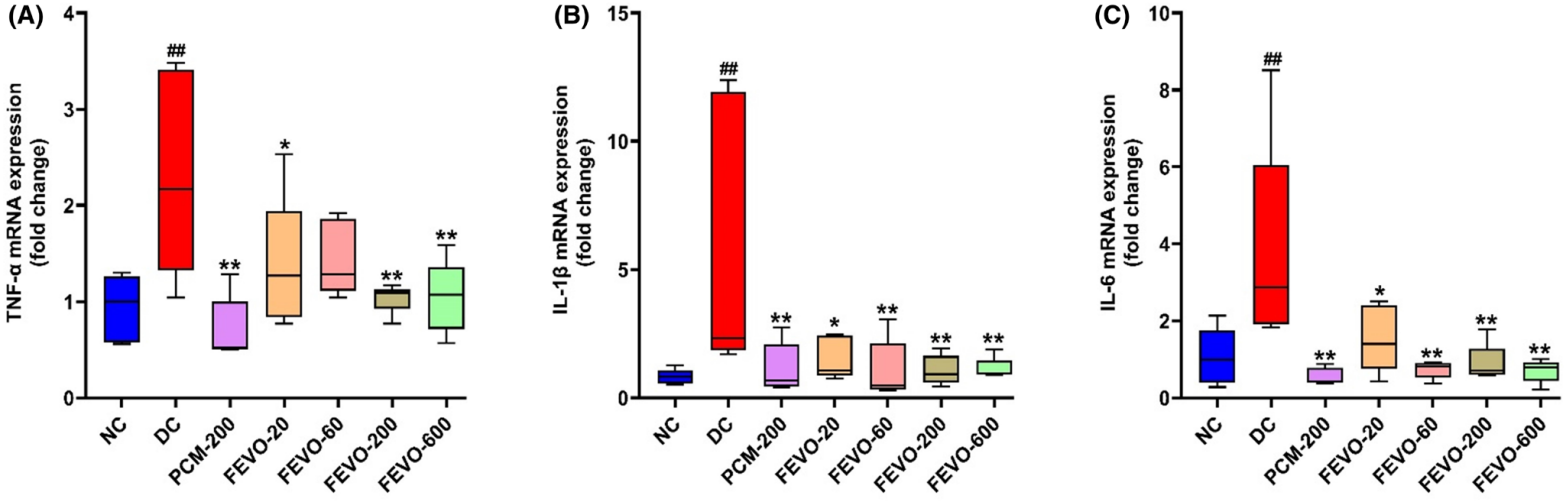
Fevogrit regulated the mRNA expression of pro‐inflammatory cytokines in the hypothalamus of LPS‐induced Wistar rats. The bar graphs (A–C) depict the effect of Fevogrit treatment on the mRNA expression of TNF‐α (A), IL‐1β (B) and IL‐6 (C) in the hypothalamus of rats, 24 h post‐LPS administration. Data are expressed as mean ± SEM (*n* = 5 animals per group) and were statistically analyzed by one‐way ANOVA followed by Dunnett's multiple comparisons test. ^##^ *p* < 0.01 vs. NC; *p < 0.05 and **p < 0.01 vs. DC.

The expression of the mRNA of pro‐inflammatory cytokines in the hypothalamus of Wistar rats was estimated 24 h post‐LPS challenge (Figure 5). It is evident that in the DC group, there was a significant increase in the gene expression of TNF‐α, IL‐1β and IL‐6, relative to the NC group (*p* < 0.01, Figure 5A–C). Paracetamol (*p* < 0.01) and Fevogrit treatment demonstrated a robust decrease in these cytokine expressions, when compared to the DC group (Figure 5A–C). A significant decrease in the mRNA expression of TNF‐α was evident, at Fevogrit doses of 20 mg/kg/day (*p* < 0.05) and 200 and 600 mg/kg/day (*p* < 0.01), respectively. Similarly, the mRNA expression of IL‐1β and IL‐6 was significantly decreased by Fevogrit (*p* < 0.05 at 20 mg/kg/day and *p* < 0.01 at 60, 200 and 600 mg/kg/day). These observations indicate that in addition to producing a peripheral response, Fevogrit also has inhibitory effects on hypothalamus pro‐inflammatory cytokines, resulting in mitigation of fever induction.

## 4 DISCUSSION

Fever is an active thermoregulatory response of the body intended to counteract any pathological threat inside the body.^4^ A polyherbal medicine, Fevogrit, has been formulated to target the fever inducing pathways and reset the thermostat in the hypothalamic area to the basal level. The therapeutic effect of any herbal formulation is the combined effect of its multiple bioactive constituents. Therefore, we have identified and quantified the marker phytocompounds present in Fevogrit, including picroside I, picroside II, vanillic acid, cinnamic acid, magnoflorine and cordifolioside A. As Fevogrit is enriched with these phytoconstituents, we hypothesized that it may be able to suppress pyrogen‐induced fever and associated inflammatory changes. In this study, we validated the potential antipyretic efficacy of Fevogrit using an endotoxin (LPS)‐induced fever model in experimental rats.

LPS is a glycolipid molecule consisting of an O‐antigen, a core polysaccharide and lipid A. When a bacterial cell invades the host body, the fragments of the membrane containing lipid A interact with toll like receptors on the surface of host immune cells. This interaction commences with the transcription of genes pertaining to endogenous pyrogens followed by fever and endotoxic shock.^16^ In the classic model of fever, LPS, when administered systemically to animals, elicits the host defense cells to release endogenous pyrogens. Following the binding of LPS to immune cells, a cascade of immune and inflammatory responses is initiated that triggers the release of endogenous pyrogens like TNF‐α, IL‐1β and IL‐6 and prompts the thermal center in hypothalamus to increase the body temperature.^17^ These pyrogens directly or indirectly modulate the thermoregulatory center in the anterior hypothalamus of the brain and consequently induce fever.^1^ In the present study also, the administration of LPS via the intraperitoneal route in rats mimicked a clinical fever and its associated inflammatory responses. The rectal temperature rose from 2 h and peaked at 6 h post‐LPS administration in the DC group, indicating an increase in the hypothalamic thermoregulatory set point. Following LPS administration, we assessed the protective effect of Fevogrit on LPS‐induced febrile responses and observed that Fevogrit substantially attenuated the LPS‐induced rise in rectal temperature, at different doses in a dose‐dependent manner (Figure 3B).

To address whether the observed antipyretic activity of Fevogrit was owing to the attenuation of the fever‐associated circulatory endogenous pyrogens, we investigated the effect of Fevogrit on the levels of circulatory pro‐inflammatory cytokines in serum. The results demonstrated that Fevogrit normalized the levels of TNF‐α, IL‐1β and IL‐6, in 6 h post‐LPS administration (Figure 4). Similarly, the mRNA expression of these cytokines was reduced significantly by Fevogrit treatment at different doses (Figure 5). Taken together, these findings suggest a possible central action of Fevogrit on the brain in inhibiting the inflammatory response and subsequent fever.

The observed pharmacological effects of Fevogrit in reducing LPS‐induced fever could be attributed to its herbal constituents and phytometabolites which are known to possess antipyretic and/or inflammation modulating activities. One of the main herbal ingredients of Fevogrit, *Tinospora cordifolia*, is renowned for its immunomodulatory effects in ancient medicinal systems, worldwide.^18^ Additionally, its antipyretic activity against the yeast‐induced pyrexia model in rats has been scientifically validated, as has its potent anti‐inflammatory activity in LPS‐stimulated monocytes and dendritic cells.^19^, ^20^ In classical Ayurvedic and Unani medicinal systems, *Swertia chirayata* has been recommended for the treatment of different types of fever.^21^ Furthermore, in Brewer's yeast‐induced pyrexia model, this medicinal plant has demonstrated significant antipyretic activity,^22^ in addition to modulating the inflammatory mediators in various in‐vivo and in‐vitro systems.^23^ Similarly, several scientific studies on *Pongamia (Millettia) pinnata* have shown its marked anti‐inflammatory activity.^24^, ^25^ *Ocimum sanctum* has been validated to reduce pyrexia and inflammation in in‐vivo animal models.^26^ Another important constituent of Fevogrit, *Picrorhiza kurroa*, has been classically described as *jvaraghna* (antipyretic) by Sage *Sushruta* in Ayurveda; and has been known for its potent antimicrobial and anti‐inflammatory properties.^27^, ^28^ Moreover, *Picrorhiza kurroa* has been found to suppress macrophage‐derived inflammatory mediators in rats.^29^ On similar lines, *Rosa centifolia* has been shown to modulate serum levels of pro‐inflammatory mediators in various experimental models of inflammation.^30^

The detected metabolites of Fevogrit picroside and magnoflorine have been shown to decrease the concentration of pro‐inflammatory cytokines in LPS‐induced acute lung injury models in mice as well as in other in‐vitro models.^31^, ^32^, ^33^ Another metabolite, vanillic acid, has been documented to attenuate neuroinflammation in the LPS‐induced neurotoxicity model in mice.^34^ Similarly, several study models have demonstrated the anti‐inflammatory and antimicrobial properties of cinnamic acid and its derivatives.^35^

The primary mechanism through which the host body responds to pathogen invasion is by releasing pro‐inflammatory cytokines from activated immune cells. Strong body of evidence suggests that these cytokines are transported from the blood to the brain where they stimulate the arachidonic acid pathway and trigger the release of central mediators like PGE2 from macrophages resident in the brain, which regulates the fever response.^2^ The current study robustly demonstrates that non‐treated LPS‐induced animals had higher levels of pro‐inflammatory cytokines including TNF‐α, IL‐1β and IL‐6 in serum and increased gene expression of these pro‐inflammatory markers in the hypothalamus. Therefore, targeting these molecular players could well be a potential therapeutic strategy to manage fever, effectively.

Development of fever following LPS stimulation is the consequence of increased peripheral as well as central inflammatory signaling. Therefore, the inflammation‐suppressive action of the bioactive components of Fevogrit could well be the primary mode of action of Fevogrit against LPS‐induced fever, as shown by the reduced levels and gene expression of TNF‐α, IL‐1β and IL‐6 in serum and hypothalamus, respectively. Consequently, this would inhibit the release of PGE2 from brain resident macrophages and therefore regulate fever.

## 5 CONCLUSION

The present study convincingly demonstrated that the oral administration of Fevogrit exerts a strong beneficial effect on the endotoxin (LPS)‐induced fever. The abundance of active anti‐inflammatory metabolites in Fevogrit and its observed inflammation suppressing effect signify that the antipyretic action of Fevogrit may stem from its peripheral inhibitory action on circulating pro‐inflammatory cytokines, as well as its central action on hypothalamus of modulating inflammatory signaling pathways responsible for setting the temperature set‐point. Thus, these findings robustly validate that Fevogrit is effective for the treatment of infection‐associated fever. Further studies are warranted to gain deeper insights into additional modes of action of Fevogrit. In addition, Fevogrit's ability to reduce fever could be investigated in other animal models, and it could be considered for detailed clinical investigations in patients suffering from fever and related etiologies.

## AUTHOR CONTRIBUTIONS

**Acharya Balkrishna**: Conceptualization, planning, visualization, supervision, writing – review and editing. **Sonam Sharma**: Conceptualization, planning, visualization, methodology, investigation, data curation, formal analysis, writing original draft. **Vivek Gohel**: Methodology, investigation, formal analysis. **Rani Singh**: Methodology, investigation, formal analysis. **Meenu Tomer**: Methodology, investigation, formal analysis, **Rishabh Dev**: Data curation, writing – review and editing, visualization, supervision. **Sandeep Sinha**: Data curation, writing – review and editing, visualization, project administration, supervision. **Anurag Varshney**: Writing – review and editing, project administration, conceptualization, visualization, supervision.

## FUNDING INFORMATION

This study was supported by internal funds from Patanjali Research Foundation Trust, Haridwar, India. No external funding was received for this research work.

## ETHICS STATEMENT

The experiments were conducted in accordance with the study protocol (wide number PRIAS/LAF/IAEC‐166) approved by Institutional Animal Ethics Committee of Patanjali Research Institute, Haridwar, India, following the guidelines of the Committee for the Control and Supervision of Experiments on Animals (CCSEA), Department of Animal Husbandry and Dairying, Ministry of Fisheries, Animal Husbandry and Dairying, Govt. of India.
